# Multiplex connectome changes across the alzheimer’s disease spectrum using gray matter and amyloid data

**DOI:** 10.1093/cercor/bhab429

**Published:** 2022-01-20

**Authors:** Anna Canal-Garcia, Emiliano Gómez-Ruiz, Mite Mijalkov, Yu-Wei Chang, Giovanni Volpe, Joana B Pereira, Alzheimer’s Disease Neuroimaging Initiative

**Affiliations:** Department of Neurobiology, Care Sciences and Society, Karolinska Institute, Stockholm, Sweden; Department of Physics, University of Gothenburg, Gothenburg, Sweden; Department of Neurobiology, Care Sciences and Society, Karolinska Institute, Stockholm, Sweden; Department of Physics, University of Gothenburg, Gothenburg, Sweden; Department of Physics, University of Gothenburg, Gothenburg, Sweden; Department of Neurobiology, Care Sciences and Society, Karolinska Institute, Stockholm, Sweden; Memory Research Unit, Department of Clinical Sciences Malmo, Lund University, Lund, Sweden

**Keywords:** Alzheimer’s disease, amyloid, gray matter, multilayer brain networks, multiplex connectome

## Abstract

The organization of the Alzheimer’s disease (AD) connectome has been studied using graph theory using single neuroimaging modalities such as positron emission tomography (PET) or structural magnetic resonance imaging (MRI). Although these modalities measure distinct pathological processes that occur in different stages in AD, there is evidence that they are not independent from each other. Therefore, to capture their interaction, in this study we integrated amyloid PET and gray matter MRI data into a multiplex connectome and assessed the changes across different AD stages. We included 135 cognitively normal (CN) individuals without amyloid-β pathology (Aβ−) in addition to 67 CN, 179 patients with mild cognitive impairment (MCI) and 132 patients with AD dementia who all had Aβ pathology (Aβ+) from the Alzheimer’s Disease Neuroimaging Initiative. We found widespread changes in the overlapping connectivity strength and the overlapping connections across Aβ-positive groups. Moreover, there was a reorganization of the multiplex communities in MCI Aβ + patients and changes in multiplex brain hubs in both MCI Aβ + and AD Aβ + groups. These findings offer a new insight into the interplay between amyloid-β pathology and brain atrophy over the course of AD that moves beyond traditional graph theory analyses based on single brain networks.

## Introduction

Alzheimer’s disease (AD) is a slowly evolving neurodegenerative disorder that usually begins with an inability to form new memories and progresses to a wide range of cognitive deficits (Winblad et al. 2016). The underlying pathological processes of AD are characterized by the abnormal accumulation of amyloid-β (Aβ) into plaques and the aggregation of tau into neurofibrillary tangles (Braak and Braak 1991). These processes are normally followed by neurodegeneration, suggesting that AD is a complex and multifactorial disease (Grothe et al. 2016).

With the development of advanced neuroimaging techniques, it is now possible to assess these diverse aspects of AD pathology in vivo. For instance, with positron emission tomography (PET), we can quantify the regional deposition of Aβ in the brain, whereas structural magnetic resonance imaging (MRI) allows measuring regional gray matter atrophy. Several studies have shown that these imaging modalities detect abnormalities in different brain areas during the course of AD (Jack et al. 2010). In addition, they have also shown that these modalities can be used to represent the brain as a complex network, formed by brain regions and their connections, known as the brain connectome (Bullmore and Bassett 2011). For instance, using structural MRI and Aβ PET, several studies have shown changes in the connectome of AD patients (Tijms et al. 2013; Pereira et al. 2016, 2018). The connectome estimated with structural MRI relies on the assessment of the regional co-variation of the mean values of cortical thickness or gray matter volumes across subjects. The assumption behind this approach is that cortical morphologic characteristics (e.g., neuronal and synaptic density, myelination level, and spatial arrangement) display similarities between connected regions (Griffa et al. 2013). Similarly, the amyloid PET connectome relies on the assessment of the regional co-variation of the mean values of PET tracer uptake across subjects (Sala and Perani 2019). In this case, increased connectivity between two regions would mean they are affected to a similar extent by amyloid pathology across different subjects. For both the structural MRI and amyloid-PET connectomes in the context of AD, the increased connectivity between brain regions is expected to be associated with the spread of gray matter atrophy and amyloid pathology, respectively.

To our knowledge, no studies have combined structural MRI and Aβ PET data within a multiplex connectome to study the interaction between gray matter atrophy and amyloid pathology in AD. A multiplex connectome is a type of multilayer network where only interlayer edges are allowed between homologous nodes (Guillon et al. 2019), which means that a region is only connected to its replicas in the different layers. The novelty of this approach is that it allows assessing the relationship between networks built using multiple neuroimaging modalities, being relevant for a disorder such as AD, which is characterized by abnormalities in multiple pathological processes (Jack et al. 2010, 2013). To our knowledge, the only studies that have assessed the multiplex connectome in AD have used functional data derived from magnetoencephalography, electroencephalography, or functional MRI (Guillon et al. 2017; Yu et al. 2017; Guillon et al. 2019; Cai et al. 2020). Combining Aβ PET and structural MRI data within a multiplex connectome in AD is important because, although both amyloid deposition and brain atrophy occur in the course of the disease (Jack et al. 2010), their relationship is not well understood, partly because these two pathological events take place at distinct points in time during the disease progression, with amyloid changes occurring first, being followed much later by brain atrophy (Bateman et al. 2012). Moreover, although the spatial topographies of amyloid deposition and brain atrophy do not overlap, there is increasing evidence showing they correlate with each other since early disease stages, including in individuals without cognitive symptoms (Harrison et al. 2021). Altogether, these lines of evidence suggest that the relationship between amyloid and atrophy in AD warrants further investigation. There are currently different multiplex network measures that can be applied to improve our understanding of this relationship. The most basic ones are the overlapping strength and the degree overlap. The overlapping strength is the sum of the connectivity strength between brain regions in the gray matter and amyloid layers and reflects how strongly the regions are connected to each other when the two layers are combined together, which is useful to understand the additive effects of amyloid and gray matter pathology in AD. The degree overlap shows which brain areas in the two layers have the exact same connections, which allows identifying regions that play a similar role across layers due to their common connectivity profile. Finally, more complex measures such as the multiplex communities identify the brain modules across the two layers, whereas the multiplex participation coefficient shows how evenly a brain region is connected in the two layers. The multiplex communities are important to understand the similarity in the brain modules present in the two layers. In contrast, the multiplex participation assesses the balance of connectivity that a brain region shows in the amyloid and gray matter layers: if there is an imbalance it means that the region is more important in one layer compared to the other; if there is a balance it means that the region can be considered as a multiplex brain hub. Finally, the multiplex clustering calculates the number of triangles formed by connections between three brain regions located in different layers, providing information about the existence of clusters that may play a role in specialized information across the two layers.

The aim of our study, which was explorative in nature, was to assess whether these different multiplex network measures could provide new insights into the interaction between amyloid pathology and gray matter atrophy across different stages of AD including cognitively normal (CN) individuals with (Aβ+) and without (Aβ−) Aβ pathology as well as cognitively impaired individuals with Aβ pathology (Aβ+) who were diagnosed with mild cognitive impairment (MCI) and AD dementia. We hypothesized that in early stages of AD (CN Aβ+), these changes would be more focalized and restricted to medial temporal regions, whereas in later disease stages these changes would be more widespread and affect areas from the parietal, occipital and frontal lobes. The novelty of our study was to move beyond traditional graph theory approaches that analyze brain networks using single imaging modalities to a more complex approach that integrates T1-weighted and ^18^F-Florbetapir PET networks within a multiplex connectome. This approach allows assessing gray matter and amyloid data together, showing whether they display a similar connectivity profile (degree overlap) and revealing their combined connectivity strengths and communities (overlapping strength, multiplex modules) as well as potential imbalances in the regional connections between the two layers (multiplex participation) or the presence of clusters of connections across layers (multiplex clustering). Importantly, this approach has only been applied in AD in a few studies but never using ^18^F-Florbetapir PET and T1-weighted data, which are interesting to analyze in this context given that, although these imaging modalities capture earlier and later changes in AD (Jack et al. 2010, 2013), respectively, they correlate with each other since very early disease stages indicating they are not independent from each other (Harrison et al. 2021).

## Materials and Methods

### Subjects

The data used in this study were obtained from the Alzheimer’s Disease Neuroimaging Initiative (ADNI2/GO), which was downloaded in 06/03/2020 (http: //adni.loni.usc.edu). Only subjects with T1-weighted and ^18^F-Florbetapir PET data that passed quality control were included. In addition, all included subjects had demographic and clinical data as well as cerebrospinal fluid levels of Aβ_42_, a well-established marker of AD pathology (Fagan and Perrin 2012).

The ADNI was launched in 2003 as a public-private partnership, led by Principal Investigator Michael W. Weiner, MD. The primary goal of ADNI has been to test whether serial MRI, PET, other biological markers, and clinical and neuropsychological assessment can be combined to measure the progression of MCI and early AD. The inclusion/exclusion criteria from ADNI are described in detail at http://www.adni-info.org/. In brief, all subjects were between the ages of 55 and 90 years, had completed at least 6 years of education and were fluent in Spanish or English. The inclusion criteria for CN subjects were Mini-Mental State Examination (MMSE) scores between 24 and 30, a Clinical Dementia Rating-Sum of Boxes (CDR-SB) score of 0, and lack of depression, MCI, or dementia. Inclusion criteria for the MCI group followed the Peterson criteria (Petersen et al. 1999) for amnestic MCI. AD participants met the National Institute for Neurological and Communicative Disorders and Stroke-Alzheimer’s Disease and Related Disorder Association (NINDS/ADRDA) criteria for probable AD, had an MMSE score between 18 and 26, and a CDR-SB of 0.5–1.0. Exclusion criteria for all participants comprised history of structural brain lesions or head trauma, significant neurological disease other than incipient AD, and the use of psychotropic medications that could affect memory.

The ADNI is conducted in accordance with the ethical standards of the institutional research committees and with the 1975 Helsinki declaration and its later amendments. Written informed consent, obtained from all subjects and/or authorized representatives and study partners, and ethical permits have been obtained at each participating site of ADNI and we have signed the data user agreements to analyze the data.

### Cerebrospinal Fluid Analysis

Lumbar puncture was performed to measure cerebrospinal fluid (CSF) Aβ_42_ levels using the fully automated Roche Elecsys-amyloid (1–42) CSF immunoassay (Bittner et al. 2016; Shaw et al. 2016). This assay is currently under development and used for research purposes only. In addition, the performance of the assay has not yet been formally established for Aβ42 concentrations <200 pg/ml or > 1700 pg/ml. None of the subjects of this study had Aβ42 concentrations <200 pg/ml and the concentrations of Aβ42 > 1700 pg/ml were replaced by 1700 pg/ml. Abnormalities in CSF Aβ42 levels were established using a previously established cut-off of CSF Aβ42 < 976.6 pg/ml (Hansson et al. 2018).

### Group Classification

Subjects were classified into 4 groups according to CSF Aβ_42_ biomarker levels and clinical diagnosis based on previous evidence showing that Aβ pathology is one of the earliest events in AD and is followed by cognitive decline and dementia (Jack et al. 2013). The groups consisted of 135 Aβ-negative CN, 67 Aβ-positive CN, 179 Aβ-positive MCI patients and 132 Aβ-positive AD patients. Patients with MCI and AD without Aβ pathology were excluded since they are not part of the AD continuum and may potentially have a non-AD disorder (Jack et al. 2018). In this study, we used CSF Aβ_42_ instead of ^18^F-Florbetapir PET levels to define Aβ status due to previous evidence showing that CSF Aβ_42_ detects earlier signs of amyloid pathology compared to amyloid-PET (Mattsson et al. 2015; Palmqvist et al. 2016; Guo et al. 2020) as well as to avoid any circularity in our analyses by not using the same variable for both group stratification and the network analyses. The total number of cases in our study with a discordant Aβ status based on CSF Aβ_42_ and ^18^F-Florbetapir PET values was 67, which corresponds to 13% of the total sample.

### Image Acquisition and Preprocessing

#### T1-Weighted MRI

All participants underwent 3 T MRI scanning using a Magnetization Prepared RApid Gradient Echo (MPRAGE) T1-weighted sequence. The T1-weighted images were preprocessed using FreeSurfer version 5.3 (https://surfer.nmr.mgh.harvard.edu). Briefly, after motion correction, removal of non-brain tissue using a hybrid watershed/surface deformation procedure (Ségonne et al. 2004) was performed, followed by automated Talairach transformation. Then, the segmentation of the subcortical white matter and deep gray matter volumetric structures was carried out (Fischl et al. 2004), followed by intensity normalization (Sled et al. 1998), tessellation of the gray matter white matter boundary, automated topology correction (Fischl et al. 2001; Ségonne et al. 2007), and surface deformation (Dale and Sereno 1993; Dale et al. 1999; Fischl and Dale 2000). The output of these preprocessing steps was visually inspected to ensure that the analyses had been carried out correctly. Thirteen subjects underwent corrections due to errors in white matter segmentation before being included in the analyses. The mean thickness of the 68 cortical regions included in the Desikan atlas (Desikan et al. 2006) in addition to the volumes of the hippocampus and amygdala (Fischl et al. 2002) were included in our analyses (Supplementary Figure 1A), similarly to a previous study (Pereira et al. 2018). To account for the influence of head size on volumetric measures, the subcortical volumes were corrected by the total intracranial volume using the following approach (Jack et al. 1989): *Adjusted Volume_i_* = *Observed Volume_i_*$-\upbeta \cdotp ( TI{V}_i-\overline{TIV})$ where $TI{V}_i$ is the *i*-th subject’s Total Intracranial Volume (TIV), $\overline{TIV}$ is the overall average TIV, and the $\beta$ is the slope of the regional volume regressed on the TIV.

#### Amyloid PET

^18^F-Florbetapir PET images acquired within 6 months from the T1-weighted images were also downloaded from the ADNI database. These images were acquired in four 5-min frames, 50–70 min after injection of approximately 10 mCi. Then, the 4 frames were coregistered, averaged and interpolated to a uniform image and voxel size (160 × 106 × 96 voxels, 1.5 mm^3^). The preprocessed PET images were then coregistered to the structural MRI scan and submitted to partial volume corrections using the PETSurfer pipeline (Greve et al. 2014, 2016) (FreeSurfer version 6.0.0, https://surfer.nmr.mgh.harvard.edu/fswiki/PetSurfer) in which the point-spread function (PSF) of 8 mm was used (Gonzalez-Escamilla et al. 2017). Mean ^18^F-Florbetapir standard uptake value ratio (SUVR) values (Supplementary Figure 1B) from the same brain regions included in T1-weighted analyses were calculated using the whole cerebellum as a reference region.

### Multiplex Network Construction

**Figure 1:**
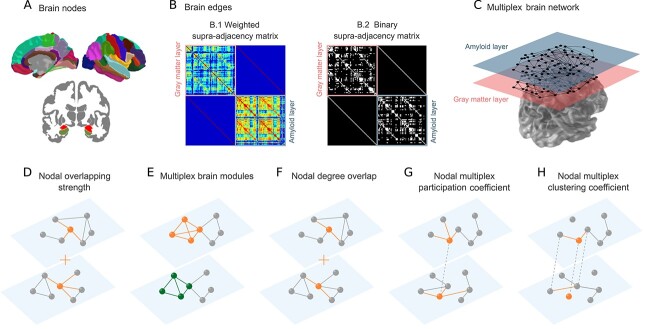
Overview of the methodology. For each group of individuals, gray matter-amyloid multiplex networks were built as a set of nodes connected by edges. The nodes were defined as the mean gray matter values for the first layer and as the mean amyloid SUVR values for the second layer of the multiplex network of 72 cortical and subcortical brain regions (*A*). The edges in both layers correspond to the partial correlation coefficients between every pair of brain regions, while adjusting for age and sex (*B*). These weighted matrices (B.1) were also binarized (B.2) using a range of densities to ensure the two layers had the same number of connections. The weighted and binary multiplex connectomes can be represented by a weighted supra-adjacency matrix (B.1) and binary supra-adjacency matrix (B.2), where the off-diagonal matrices are the connections between layers (inter-layer) and the diagonal matrices are the intra-layer matrices containing the structural and the amyloid edges, respectively. The intra-layer adjacency matrices were then integrated into a multiplex network (*C*). The nodal overlapping strength (*D*) and the multiplex brain modules (*E*) were calculated in the weighted multiplex networks, whereas the nodal degree overlap *(F*), the multiplex participation coefficient (*G*), and the multiplex clustering coefficient (*H*) were calculated in the binary multiplex networks.

For each group, we constructed a multiplex network with two layers: one with the gray matter network and the other with the amyloid network. Each network was built as a collection of nodes representing brain regions connected by edges. In the gray matter network, the nodes were defined using the mean cortical thickness or subcortical volumes of 72 brain regions, whereas in the amyloid network the nodes were defined using the mean SUVR values of the same brain regions (Figure 1A). For each network, the edges were calculated as the partial correlation coefficients between every pair of brain regions using Pearson’s R (Figure 1B), while controlling for the effects of age and sex. All self-connections were ignored. Then the gray matter layer and the amyloid layer were integrated into a multiplex network (Figure 1C). This multiplex network can be represented in a weighted supra-adjacency matrix $\mathcal{W}$ (Figure 1B.1), which is given by the intra-layer adjacency matrices on the main diagonal $\mathcal{W}$ = $\Big\{{w}_{ij}^{[\alpha]}\Big\}$, where ${w}_{ij}^{[\alpha]}$ is the weight of the edge between nodes *i* and *j* in layer α = 1, 2. Layer 1 corresponds to the gray matter layer and layer 2 to the amyloid layer. Both layers have *N* = 72 nodes. Since most graph theory measures are influenced by the number of connections (Fornito et al. 2013), in addition to conducting the network analyses on weighted connectivity matrices, we also performed the analyses on matrices that were binarized using a range of densities to ensure that the two layers and all groups had the same number of connections (represented in Figure 1B.2).

### Multiplex Network Analysis

#### Weighted Analysis

We calculated the overlapping strength (Figure 1D) and the multiplex communities (Figure 1E) using the weighted supra-adjacency matrix $\mathcal{W}$. The overlapping strength is the sum of the connectivity strength of a node in both layers (Figure 1D), defined as:

(1) \begin{equation*} {s}_i={s}_i^{\left[1\right]}+{s}_i^{\left[2\right]} \end{equation*}

where ${s}_i^{[1]}$ and ${s}_i^{[2]}$ are the strength of node *i* in layer 1 and layer 2, respectively.

In order to calculate the multiplex communities, we used a multilayer version of the modularity quality function (Mucha et al. 2010), which can be calculated for two layers as:

(2) \begin{eqnarray*} Q\!\!\!\!\!\!\!\!\!\!&&=\frac{1}{\mu}\sum_{i,j=1}^N\left[\left({w}_{ij}^{\left[1\right]}+{w}_{ij}^{\left[2\right]}\right)-\gamma \left({w}_{0, ij}^{\left[1\right]}+{w}_{0, ij}^{\left[2\right]}\right)+2\omega{\delta}_{ij}\right]\nonumber\\ &&\quad\times\,\delta \left({g}_i^{\left[1\right]}+{g}_j^{\left[1\right]}\right)\delta \left({g}_i^{\left[2\right]}+{g}_j^{\left[2\right]}\right) \end{eqnarray*}

where *μ* is the total weights of the edges, γ is the resolution parameter, ${w}_{0, ij}^{[1]}$ and ${w}_{0, ij}^{[2]}$ are the weights of the associated null matrices for layer 1 and layer 2, respectively, ω is the inter-layer coupling parameter, ${\delta}_{ij}$= 1 if *i* = *j* and 0 otherwise, ${g}_i^{[1]}$ and ${g}_i^{[2]}$ are the community assignment of node *i* at layer 1 and layer 2, respectively, ${g}_j^{[1]}$ and ${g}_j^{[2]}$ are the community assignment of node *j* at layer 1 and layer 2, respectively, and $\delta \Big({g}_i^{[1]}+{g}_j^{[1]}\Big)$ = 1 and $\delta \Big({g}_i^{[2]}+{g}_j^{[2]}\Big)$ = 1 if the community assignments ${g}_i^{[1]}$, ${g}_j^{[1]}$ and ${g}_i^{[2]}$, ${g}_j^{[2]}$ of nodes *i* and *j* are the same and 0 otherwise.

The null models ${W}_0^{[1]}=\Big\{{w}_{0, ij}^{[1]}\Big\}$ and ${W}_0^{[2]}=\Big\{{w}_{0, ij}^{[2]}\Big\}$ are obtained by randomizing the edges of each layer, while maintaining the layer node’s strength (Newman and Girvan 2004; Newman 2006). By varying the resolution parameter γ, we can control the size and number of the detected communities (or modules). Low values of γ produce fewer but larger communities, while high values of γ produce many smaller communities. The parameter *ω* controls the weights of the edges between layers. Small values of *ω* will highlight the unique modular structure of each layer, while larger values highlight the shared modular structure across layers. We computed the multiplex communities using different combinations of resolution parameters {0.5, 0.8, 0.9, 1, 1.1, 1.2, 2} and inter-layer parameters {0.25, 0.5, 0.75, 1}. Then, we calculated the normalized Variation of Information (VI) (Meilă 2007), which is a measure of distance between two community partitions, to identify the most optimal parameters (Supplementary Figure 2). We used the code of *partition_distance* from BCT (Rubinov and Sporns 2010) to calculate the normalized VI matrix. Based on this matrix, the most optimal parameters in our study were a resolution parameter γ = 1 and an inter-layer parameter *ω* = 1, which also led to a coherent number of modules. To optimize the multilayer modularity, we used the code of the generalized version of the Louvain algorithm implemented in MATLAB provided by the genlouvain package (Jeub et al. 2011-2019). Using this algorithm, the multilayer communities are obtained by maximizing the multilayer modularity through several iterations until the most optimal and stable module partition is found. The result of this measure is a number for each node that indicates the community assignment in the gray matter layer and another number that indicates the community assignment in the amyloid layer. In that way, the communities of each layer are comparable, because they come from the same modularity optimization.

Finally, we also computed the persistence of the multilayer communities obtained for each group as the normalized sum of the number of nodes that belong to the same community in the two layers (Jeub et al. 2011-2019). The persistence varies between 0 (no persistence) and 1 (high persistence).

#### Binary Analysis

To compare topological measures in the multiplex connectome between groups we used the binary supra-adjacency matrix $\mathcal{A}=\Big\{{A}^{[1]},{A}^{[2]}\Big\}$. The adjacency matrices ${A}^{[1]}$ and ${A}^{[2]}$ were obtained from the weighted supra-adjacency matrix $\mathcal{W}=\Big\{{W}^{[1]},{W}^{[2]}\Big\}$ by binarization (Figure 1B.2, binary matrix) using a range of network densities D to ensure all groups had the same number of edges in both layers: *D_min_* = 2% to *D_max_* = 30%, in steps of 1%. The minimum density (2%) was chosen to ensure that the number of edges was higher than the number of nodes and therefore avoid widely disconnected layers. The maximum density (30%) was selected to ensure a small-world index >1 (Supplementary Table 1), which is typical of networks with a biologically meaningful organization (Bullmore and Sporns 2012; Muldoon et al. 2016).

The network topology can be assessed using a variety of measures that characterize the centrality, the integration, and the segregation of a network.

In this study, we calculated the degree overlap (Figure 1F) as a measure of multiplex centrality. This measure provides the overlapping connections of the multiplex network, which are the number of edges connected to a node *i* in both layers. It is defined as:

(3) \begin{equation*} {d}_i=\sum_{j=1}^N{a}_{ij}^{\left[1\right]}{a}_{ij}^{\left[2\right]} \end{equation*}

where ${a}_{ij}^{[1]}$ is the link between node *i* and *j* in layer 1 and ${a}_{ij}^{[2]}$ is the link between node *i* and *j* in layer 2.

We also evaluated the integration between layers by calculating the nodal multiplex participation coefficient (Figure 1G). The nodal multiplex participation coefficient ${p}_i$ measures how evenly a node *i* is connected to the different layers of the multiplex network: higher values of ${p}_i$ can be found in a node that has a similar participation coefficient in the different layers of the multiplex network. Moreover, nodes with high ${p}_i$ are considered central or multiplex hubs as they would allow for a better exchange of information between different layers. The multiplex participation coefficient of node *i* is defined as (Battiston et al. 2014):

(4) \begin{equation*} {p}_i=\frac{M}{M-1}\left[1-\sum_{\alpha =1}^M{\left(\frac{k_i^{\left[\alpha \right]}}{o_i}\right)}^2\right] \end{equation*}

where *M* is the number of layers, ${k}_i^{[\alpha ]}$ is the degree of node *i* at the *α*-th layer and ${o}_i={\sum}_{\alpha }{k}_i^{[\alpha ]}$ is the overlapping degree of node *i*. If the node *i* has the same degree in all layers, ${p}_i$ is equal to 1. In contrast, if the degree of node *i* is different from zero in only one layer, ${p}_i$ is equal to 0.

Finally, we evaluated the segregation between layers by calculating the multiplex clustering coefficient (Figure 1H), a measure that reflects the presence of triangles (number of neighbors of a node that are also neighbors of each other) between the different layers. In multiplex networks, an α-triad (α-triangle) is defined as a triad (triangle), which uses edges from α different layers. For each node *i*, the multiplex clustering coefficient ${c}_i$ is defined as the ratio between the number of 2-triangles with a vertex in *i* and the number of 1-triads centered in *i* (Battiston et al. 2014):

(5) \begin{equation*} {c}_i=\frac{\sum_{\alpha }{\sum}_{\beta \ne \alpha }{\sum}_{i\ne m,j}{\left({a}_{ij}^{\left[\alpha \right]}{a}_{jm}^{\left[\beta \right]}{a}_{mi}^{\left[\alpha \right]}\right)}^{\raisebox{1ex}{$1$}\!\left/ \!\raisebox{-1ex}{$3$}\right.}}{\left(M-1\right){\sum}_{\alpha }{k}_i^{\left[\alpha \right]}\left({k}_i^{\left[\alpha \right]}-1\right)} \end{equation*}

where ${a}_{ij}^{[\alpha]}$ is the link between node *i* and *j* in layer *α*. Thus, ${c}_i$ quantifies the fraction of triangles where edge *jm* belongs to layer *β*, while the other two edges *ij* and *im* belong to layer α.

The construction of multiplex brain networks and graph theory analysis were performed using an adapted version of Brain Analysis using Graph Theory (BRAPH (Mijalkov et al. 2017), http://braph.org/).

### Statistical Analysis

To assess differences between groups in demographic, clinical and genetic variables, the Kruskal–Wallis rank sum test was applied due to the non-normal distribution of the data using R Studio (version 4.0.3).

To assess the statistical significance of the differences in the network measures between groups, we carried out nonparametric permutation tests with 10 000 replicates (Bassett et al. 2008; He et al. 2008). First, we calculated the difference of the network measures values between every pair of groups. In the binary analyses, this was done at each network density (2%–30%). Then, we obtained an empirical distribution of the difference by randomly reallocating all the values into 2 groups and recalculating the mean differences between the 2 randomized groups. This randomization procedure was repeated 10 000 times and the 95th confidence intervals of the resulting distribution were used as the critical values for a 2-tailed test at *P* < 0.05. The *P*-values of the 72 brain regions obtained from the group comparisons in the binary analyses were averaged across densities. Finally, false discovery rate (FDR) corrections (Benjamini and Hochberg 1995) were applied across the 72 brain regions to control for multiple comparisons (*q* < 0.05). The multiplex analyses results were visualized with ggseg, a Visualization for Brain Statistics R-package (Mowinckel and Vidal-Piñeiro 2019), using the two available atlases, the Desikan-Killany atlas (aparc) and the automatic subcortical segmentation atlas (aseg).

### Data Availability

All the data used in the current study were obtained from ADNI, an open-access multicenter cohort that anyone can apply for. The code that was used in this study will be made available through our freeware graph theory software BRAPH, and can be downloaded from the following website: http://braph.org/.

## Results

The characteristics of the sample can be found in Table 1. Age, sex, education, MMSE, APOE ϵ4, CSF Aβ_42_, and the percentage of amyloid-PET-positive cases were compared between all groups with the Kruskal–Wallis rank sum test.

**Table 1:** Characteristics of the sample and values represent medians followed by the interquartile range for each group, unless otherwise specified

	CN Aβ− (*n* = 135)	CN Aβ + (*n* = 67)	MCI Aβ + (*n* = 179)	AD Aβ + (*n* = 132)	*P-*value
Age (years)	73 (9.55)	72.9 (8.1)	74.2 (9)	74.6 (11.18)	0.720
Sex (f/m)	73/62	40/27	76/103	55/77	0.019
Education (years)	17 (3)	17 (2)	16 (4)	16 (4)	0.008
MMSE	29 (2)	29 (1)	28 (3)	23 (4)	<0.001
APOE ϵ4 (%)	20	44.8	62	72	<0.001
CSF Aβ_42_ (pg/ml)	1587 (399.5)	752.9 (322.95)	686.1 (283.4)	585.2 (233.7)	<0.001
Amyloid-positive PET (%)	12.59	74.63	83.24	97.73	<0.001

As expected, the MCI and AD patients had worse MMSE scores and the prevalence of the APOE ϵ4 allele was higher in the Aβ-positive compared to the Aβ-negative groups.

### Multiplex Weighted Analysis

#### Nodal Overlapping Connectivity Strength

**Figure 2:**
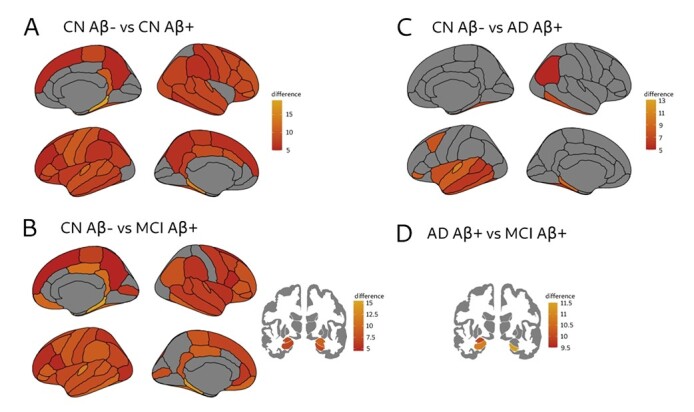
Significant differences between groups in the nodal overlapping strength. There were significant overlapping strength increases in CN Aβ + compared to the CN Aβ− group (*A*), in MCI Aβ + compared to the CN Aβ− group (*B*), in AD Aβ + compared to the CN Aβ− group (*C*) and in MCI Aβ + compared to the AD Aβ + group (*D*). Lighter orange indicates larger increases. All results were adjusted for multiple comparisons using FDR corrections at *q* < 0.05.

We observed higher overlapping connectivity strength in CN Aβ + (Figure 2A) and MCI Aβ + groups (Figure 2B) compared to the CN Aβ− group in widespread brain areas, with the greatest differences being found in the parahippocampal gyri and temporal poles. In contrast, AD Aβ + patients showed less widespread increases in this measure compared to the CN Aβ− group, with the main changes being observed in left temporal areas but also a few parietal and frontal regions (Figure 2C). MCI Aβ+ patients also showed a higher overlapping strength in the hippocampi and left amygdala compared to AD Aβ+ patients (Figure 2D).

Overall, these results showing greater overlapping strength changes in the CN Aβ+ and MCI Aβ+ individuals compared to the other groups were due to the overall greater connectivity strength between brain areas in their amyloid connectivity matrices (Supplementary Figure 3), which when summed to the connectivity strength in the gray matter matrices gave place to higher multiplex overlapping strength in these two groups. There were no significant differences between AD Aβ+ and CN Aβ+ or between MCI Aβ+ and CN Aβ+.

#### Multiplex Brain Modules

**Figure 3:**
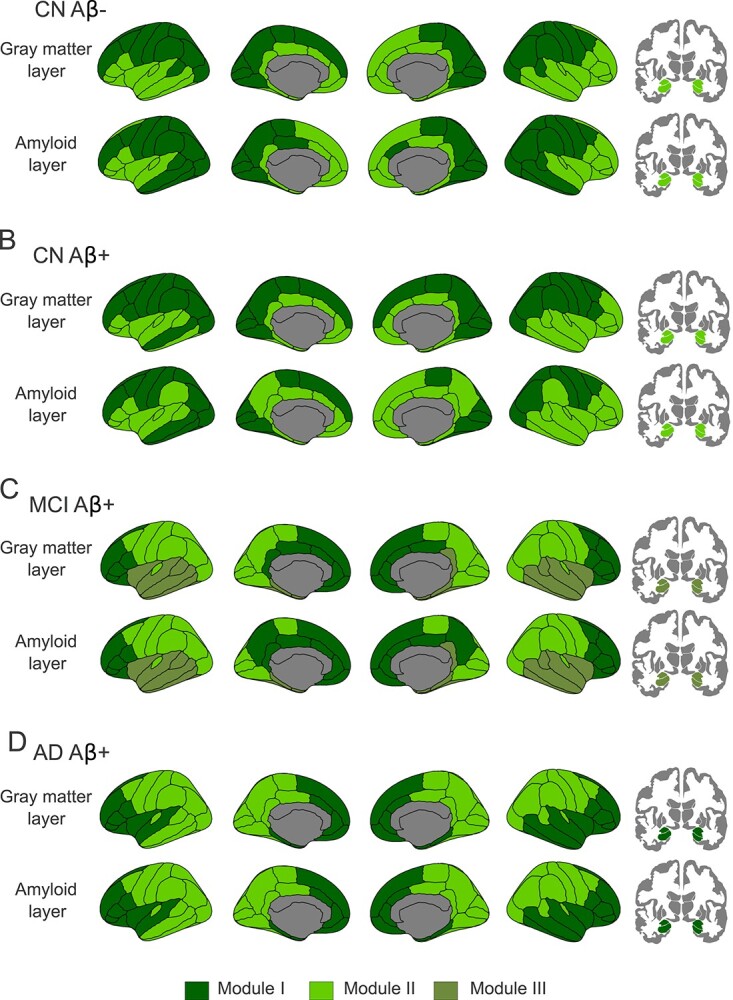
Multiplex brain modules for each group. We found 2 modules in the CN Aβ− group (*A*), 2 modules in the CN Aβ + group (*B*), 3 modules in the MCI Aβ + group (*C*), and 2 modules in the AD Aβ + group (*D*).

We identified 2 modules in the AD Aβ+, CN Aβ+ and CN Aβ− groups, whereas the MCI Aβ + group presented 3 modules (Figure 3).

In the CN Aβ− group (Figure 3A), module I was the largest and included frontal, parietal, occipital and lateral temporal regions, whereas module II included areas of the limbic system such as the cingulate gyrus, insula, medial temporal areas, amygdala, and hippocampus. Differences in the modules between the two layers were observed only in a few regions, with module II including more temporal areas in the gray matter layer. The persistence of the multiplex modules in this group was 0.88.

In the CN Aβ+ group (Figure 3B), the modules showed a similar pattern. For instance, module I included frontal, parietal, occipital and left lateral temporal regions whereas, module II included limbic regions in both layers. Differences in the modules between the two layers were observed only in a few regions, with module II including more parietal areas in the amyloid layer. The persistence of the multiplex modules in CN Aβ+ was 0.85.

In the MCI Aβ+ group (Figure 3C), there were three clearly defined modules. Module I included anterior frontal, medial frontal and medial parietal regions, whereas module II included sensorimotor, parietal and occipital areas and module III included temporal regions. The only difference between the modules in the two layers was found in the precuneus, which belonged to the amyloid but not the gray matter layer in module I. The persistence in this group was the highest and equal to 0.97.

Finally, in the AD Aβ+ group (Figure 3D), module I included anterior frontal, medial parietal, and areas of the limbic system, whereas module II comprised sensorimotor, parietal, middle temporal, and inferior temporal regions. There were differences in modules composition in the frontal pole, belonging to module I in the gray matter layer and to module II in the amyloid layer. Similar to the MCI Aβ + group, the persistence of the AD Aβ + modules was equal to 0.97.

### Multiplex Binary Analysis

#### Nodal Degree Overlap

**Figure 4:**
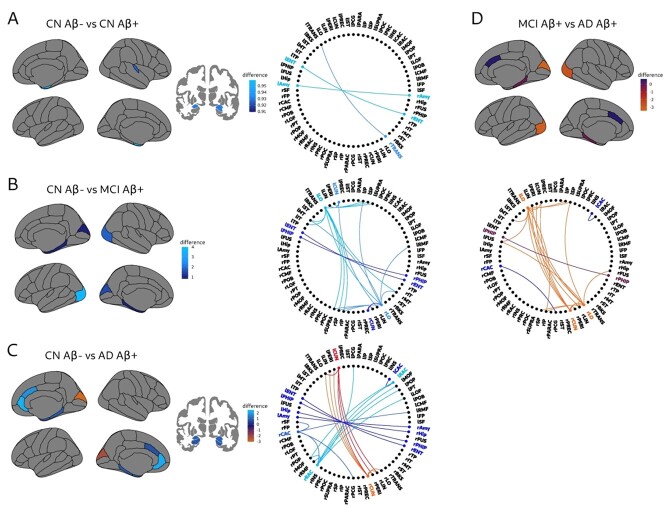
Significant differences between groups in the nodal degree overlap. Significant differences between CN Aβ− and CN Aβ + (*A*), CN Aβ− and MCI Aβ + (*B*), CN Aβ− and AD Aβ + (*C*), and between MCI Aβ + and AD Aβ + (*D*). Differences in (A) were due to a loss of connections in the entorhinal cortex, the amygdala and the right transverse temporal; differences in (B) were due to a loss of connections in the entorhinal cortex and the parahippocampal gyri in addition to several connections between occipital, parietal, and temporal areas; differences in (C) were due to a loss of connections between temporal areas and frontal areas and increase of connectivity between occipital areas; and differences in (D) were due to the increased connectivity between occipital, temporal and parietal areas in addition to the loss of connectivity between the caudal anterior cingulate, the right posterior cingulate, and the left rostral anterior cingulate. These changes in the connections can be observed in the respective connectograms on the right. Lighter blue indicates larger decreases in the Aβ + groups compared to the CN Aβ− group, and lighter red-orange indicates increases in the AD Aβ + compared to the CN Aβ− and compared to the MCI Aβ+. All results were adjusted for multiple comparisons using false discovery rate (FDR) corrections at *q* < 0.05.

The CN Aβ + group showed a lower nodal degree overlap compared to CN Aβ− subjects in the bilateral entorhinal cortex, the right transverse temporal, and the bilateral amygdala (Figure 4A). These results were driven by a loss of connectivity between the left side of these regions and the right side in the two layers in CN Aβ + subjects, as can be observed in the connectogram.

In addition, we found significant degree overlap decreases in the bilateral entorhinal cortex, bilateral parahippocampal gyri, bilateral lateral occipital gyri, and bilateral cuneus in the MCI Aβ + compared to the CN Aβ− group (Figure 4B). These decreases were due to a loss of connections between the left and right entorhinal cortex, the left and right parahippocampal gyrus, and several connections between occipital, parietal, and temporal areas.

Similarly to MCI Aβ+, AD Aβ + patients also showed degree overlap decreases (Figure 4C) in the bilateral entorhinal cortex, parahippocampal gyrus, anterior cingulate, amygdala, and hippocampus in addition to increases in the bilateral cuneus compared to the CN Aβ− group. These results were driven by a loss of connectivity between temporal areas and frontal areas and increases of connectivity between occipital areas.

Finally, AD Aβ + patients showed degree overlap decreases in the bilateral caudal anterior cingulate (Figure 4D) as well as increases in the bilateral lateral occipital gyri, the bilateral parahippocampal gyri, and the right cuneus compared to MCI Aβ+. The decreases in the bilateral caudal anterior cingulate were driven by the loss of connections between this region, the right posterior cingulate, and the left rostral anterior cingulate, whereas the increases were due to higher connectivity between occipital, temporal, and parietal areas.

There were no significant differences between AD Aβ + and CN Aβ + or between MCI Aβ + and CN Aβ+. The regional values of the degree overlap in each group can be found in Supplementary Figure 4.

#### Nodal Multiplex Participation Coefficient

**Figure 5:**
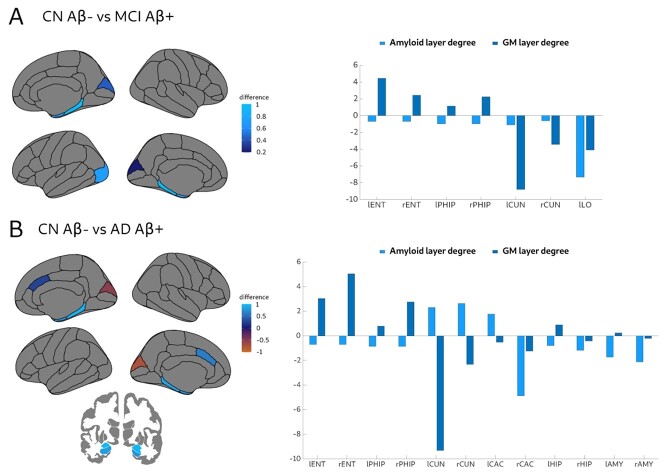
Significant differences between groups in the nodal multiplex participation coefficient. Significant differences between CN Aβ− and MCI Aβ + (*A*), and between CN Aβ− and AD Aβ + (*B*), which were mainly due to an imbalance of connectivity in the two layers as can be seen on the right plots. Lighter blue indicates larger decreases in MCI and AD groups, while lighter red indicates increases in AD. All results were adjusted for multiple comparisons using false discovery rate (FDR) corrections at *q* < 0.05.

We found that the MCI Aβ + group showed a lower nodal multiplex participation coefficient (Figure 5A) in the bilateral cuneus, parahippocampal, and entorhinal areas as well as the left lateral occipital gyrus compared to CN Aβ− subjects. These results were driven by an imbalance of connectivity in the two layers. Specifically, there was a higher connectivity in the entorhinal cortex and parahippocampal gyri in addition to lower connectivity in the cuneus and left lateral occipital gyri in the gray matter layer with respect to the amyloid layer in MCI Aβ +.

The AD Aβ + group also showed a lower nodal multiplex participation coefficient (Figure 5B) in the entorhinal cortex, parahippocampal gyrus, hippocampus, amygdala and the caudal anterior cingulate, and higher in the cuneus. These results were due to the temporal regions being more connected in the gray matter layer, whereas the anterior cingulate and cuneus regions were more connected in the amyloid layer in AD Aβ + patients.

There were no significant differences between AD Aβ + and MCI Aβ+, AD Aβ + and CN Aβ+, MCI Aβ + and CN Aβ + as well as CN Aβ + and CN Aβ− groups.

#### Nodal Multiplex Clustering Coefficient

No significant differences were found in the multiplex clustering coefficient between any of the groups.

## Secondary Analyses Using Cortical Volume Measures in the Multiplex Network Analyses

We also conducted additional analyses using cortical volumes instead of cortical thickness to build the multiplex networks and compared them between groups (Supplementary Figure 5). These analyses showed similar, albeit less widespread results, compared to the ones with cortical thickness, in line with previous evidence showing that thickness measures are more sensitive to gray matter changes occurring in AD compared to volume measures (Dickerson et al. 2009). Moreover, although the multiplex communities using volume data were less well defined compared to the ones with thickness, there was still a bilateral temporal module in the MCI Aβ + group in the volume-amyloid multiplex networks.

## Discussion

In this study, we examined the relationship between gray matter atrophy and amyloid deposition across different stages of AD using a multiplex network approach. Our findings revealed widespread increases in the overlapping strength and decreases in the degree overlap in all Aβ-positive individuals. In addition, there was a reorganization of the brain communities in the MCI Aβ + group and an imbalance in the number of connections between the gray matter and amyloid layers in both MCI Aβ + and AD Aβ + patients. These findings indicate that multiplex networks can be used to characterize different stages of AD and provide information on how amyloid pathology and gray matter interact as the disease progresses. Below we discuss these findings in detail.

There is increasing evidence showing that the progression of amyloid Aβ pathology and gray matter atrophy in AD follows a characteristic spatial pattern (Braak and Braak 1991; Thal et al. 2002; Thompson et al. 2003; Singh et al. 2006; Sepulcre et al. 2013; Grothe et al. 2016; Sepulcre et al. 2016). In the case of amyloid pathology, it usually appears in neocortical regions and then spreads to the entorhinal cortex and other limbic areas (Braak and Braak 1991; Thal et al. 2002). In contrast, gray matter atrophy usually appears in the entorhinal cortex and hippocampus in early AD, being followed by atrophy in medial and lateral parietal areas, frontal brain regions, and the sensorimotor cortex as the disease progresses (Thompson et al. 2003; Singh et al. 2006). In this study, we analyzed the relationship between these two pathological events using multiplex brain networks. We used a group of control subjects that are amyloid-negative as a reference group for the comparisons of the groups with amyloid-β pathology. Although in this group there was no substantial gray matter atrophy and amyloid deposition, there was sufficient variability in the amyloid and gray matter signals to conduct a network analysis (Supplementary Figure 1). Furthermore, our study is not the first one to measure network topology in amyloid-negative individuals (Pereira et al. 2018; Kim et al. 2019) and there are a lot of studies studying gray matter covariance networks in older healthy subjects (Montembeault et al. 2012), even without amyloid pathology (Tijms et al. 2016; Voevodskaya et al. 2018; Dicks et al. 2020).

In this study, we found widespread increases in the overlapping strength in CN Aβ + and MCI Aβ + subjects, whereas in AD Aβ + patients, these changes were mainly confined to the temporal lobes. The overlapping strength is the sum of the connectivity strengths of the two layers, and it was higher in CN Aβ + and MCI Aβ + subjects due to their amyloid layers being more strongly connected than the other groups. In addition, AD Aβ + patients showed a lower overlapping strength in the hippocampi and left amygdala compared to the MCI Aβ + subjects. This finding is most likely due to the fact that amyloid deposition rises rapidly in early and mild stages of the disease in CN and MCI subjects with amyloid pathology, before reaching more stable levels in late disease stages in patients with AD dementia (Jack et al. 2010, 2013).

In contrast to the overlapping strength, we found that the multiplex communities identified changes mainly in MCI Aβ + individuals. In fact, this measure, which reflects the communities or subnetworks in the amyloid and gray matter layers, showed an interesting re-organization in this group who had three modules instead of two compared to the other groups. This third module was composed by temporal brain regions, which is in line with previous studies showing a correlation between amyloid deposition and gray matter loss in temporal areas, which might also be important points of interaction between amyloid pathology and tau pathology, which is known to be one of the drivers of gray matter atrophy in AD (Sepulcre et al. 2016). Thus, the emergence of a temporal community in the amyloid and gray matter layers might signal the transition from MCI to AD dementia.

We also assessed topological measures associated with centrality, integration, and segregation. The degree overlap is a centrality measure that increases when a brain region has the same number of connections in the two layers. We found significant decreases in the degree overlap mainly in temporal regions such the entorhinal cortex, hippocampus and transverse temporal gyri in CN Aβ + subjects, which was due to a loss of connections between the left and the right side of these regions. Moreover, similar results were also found in the MCI Aβ + group, which showed additional decreases in occipital regions due to a widespread loss of connections between these areas and the rest of the network, in line with the more advanced disease stage of this group. Finally, in AD Aβ + patients, we found decreases in the anterior cingulate and parahippocampus, which were mainly driven by a loss of connections between bilateral frontal and limbic areas. However, interestingly, AD Aβ + patients also showed increases in the degree overlap in the cuneus, which became more connected to other occipital areas. These increases in occipital brain connectivity could still be observed when the AD Aβ + patients were compared to MCI Aβ + subjects and could potentially reflect the more prominent role occipital regions play in later disease stages by being connected to the same brain areas in different layers. The biological interpretation behind increased connectivity in gray matter covariance networks in AD is usually attributed to the fact that if two regions get atrophied at the same rate across different individuals, the correlation coefficients between those regions will increase due to shared mechanisms in neurodegeneration. Similarly, increases of connectivity in amyloid covariance networks are usually attributed to the fact that if two regions are similarly affected by amyloid pathology, their connectivity will also increase. Thus, altogether these results indicate there is a general decrease in the overlap of connections between the amyloid and gray matter layers in the amyloid-positive groups. The observed loss of connections might be due to a disconnection process occurring in one or both the gray matter and amyloid layers. For instance, in the case of medial brain areas, we observed a lower number of connections in the gray matter layer in MCI Aβ + and AD Aβ + groups compared to the amyloid layer. These results are in line with the hypothesis that AD is a disconnection syndrome (Delbeuck et al. 2003). However, here we show that this disconnection can also be captured using measures of covariance in gray matter and amyloid, which show that in early disease stages (CN Aβ + subjects) this is due to a loss of connections between the same brain regions in the left and right hemispheres, whereas in later disease stages (MCI Aβ + and AD Aβ+), this is due to a decrease as well as increase of several connections between widespread brain areas. These findings illustrate the complexity of later disease stages, which are characterized by changes of connectivity in both directions, when both gray matter atrophy and amyloid deposition are affecting widespread areas. The multiplex participation coefficient is an integration measure that increases with the number of direct paths in the two layers. It can be used to detect highly central regions such as the multiplex hubs, which have a high number of direct connections in both layers. In this study, we found a loss of multiplex brain hubs in later stages of AD. Specifically, we found that the MCI Aβ + patients had a lower nodal multiplex participation coefficient in lateral occipital, entorhinal and parahippocampal areas, whereas AD Aβ + patients showed lower multiplex participation coefficients in the anterior cingulate, temporal pole, entorhinal cortex, hippocampus and amygdala in addition to increases in the cuneus in comparison with the CN Aβ− group. In general, the differences between these groups occurred due to an imbalance in the number of connections between layers. In the MCI Aβ + group, this imbalance was driven by a higher connectivity in temporal regions and lower connectivity in occipital regions in the gray matter layer with respect to the amyloid layer. A similar scenario was observed in the AD Aβ + group, in which temporal regions were more connected in the gray matter layer, whereas the cuneus and left anterior cingulate regions were more connected in the amyloid layer. These differences might be due to the more prominent role that temporal regions play in brain atrophy in the course of AD (Thompson et al. 2003; Singh et al. 2006; Sepulcre et al. 2016), whereas neocortical regions such as the anterior cingulate play a more important role in amyloid pathology progression (Braak and Braak 1991; Thal et al. 2002; Sepulcre et al. 2013).

Finally, in this study we also analyzed segregation with the multiplex clustering coefficient, a measure that calculates the number of triangles between the two layers. Although this measure has been shown to be useful to identify properties of local information processing across time and across frequencies using EEG data (Cai et al. 2020), in our case, no significant differences were found between any of the groups. Thus, it is possible that this measure might be better suited to assess segregation changes within the same imaging modality. An alternative explanation for the lack of multiplex clustering results would be that segregation measures are less sensitive to changes occurring in amyloid and gray matter covariance networks in AD, which would be in line with the results of previous studies showing that measures of centrality are more useful to characterize the amyloid network (Pereira et al. 2018), and the fact that gray matter covariance networks have shown contradictory clustering results, showing both clustering decreases and increases in AD with respect to controls (Tijms et al. 2013).

Despite the value of our study in characterizing different stages of AD using a novel multiplex network approach, some limitations should be recognized. First of all, we used cross-sectional imaging data to perform the multiplex analyses, which did not permit us to assess how amyloid accumulation and gray matter atrophy change over time in each group. Another limitation is the fact that only two imaging modalities were included, amyloid PET and structural MRI, because we were interested in assessing how the network topologies of two biological processes that become altered in earlier and later AD stages were related in the highest possible number of brain images acquired within 6 months from each other. Future studies should include additional imaging modalities such as FDG-PET in the multiplex analyses to understand the interplay between amyloid pathology and brain atrophy with other pathological processes occurring in AD. It would also be interesting to assess the relationship between our multiplex layers with tau pathology since tau has been shown to be closely associated both with amyloid pathology and gray matter atrophy. An additional limitation is the fact that structural covariance networks do not allow calculating multiplex measures for each subject but only per group, and therefore it is not possible to correlate the multiplex results with the cognitive and clinical measures of single individuals or to perform statistical comparisons with the multiplex communities, which would have been very interesting. Moreover, like in all graph theory studies using structural MRI or static PET data, the underlying mechanisms for correlations in gray matter or amyloid pathology is not entirely clear, although increasing evidence suggests that they reflect shared neurodegeneration or pathological mechanisms between brain areas (Lerch et al. 2006; Zhu et al. 2012; Alexander-Bloch et al. 2013). Finally, we also did not compare our results with other available methods that integrate multimodal imaging data such as sparse inverse covariance estimation. Using this method, Li et al. (2018) previously fused T1-weighted, amyloid PET, and FDG-PET data into group covariance matrices in CN, MCI, and AD patients and found significant decreases in connectivity within the temporal lobe as well as decreases of connectivity between the parietal and occipital lobes, the occipital and temporal lobes, and the parietal and temporal lobes across different groups. The novelty of our study with respect to Li et al. (2018) is that we did not fuse different imaging modalities into the same connectivity matrix but instead integrated them as two separate layers in the same network. Our approach therefore assesses how amyloid and gray matter interact as independent processes instead of merging the information provided by each of them into the same connectivity matrix, allowing to compute a range of multiplex network measures. Despite all these limitations, our study also has some methodological strengths such as the fact that we applied partial volume corrections to the amyloid PET data in order to avoid confounding effects associated with brain atrophy in AD (Thomas et al. 2011). This approach has been recently shown to be particularly useful in the assessment of amyloid PET covariance networks using graph theory, improving the interpretability of the results and being more sensitive to differences between diagnostic groups due to an optimized characterization of network efficiency and modularization (Gonzalez-Escamilla et al. 2021).

To conclude, in this study, we show that multiplex network analyses are useful to detect differences across different stages of AD, with the overlapping connectivity strength being sensitive to changes occurring in CN Aβ + and MCI Aβ + subjects due to the more dynamic changes their amyloid covariance networks are undergoing, whereas the multiplex communities revealed the emergence of a third temporal module in MCI Aβ + subjects, likely reflecting the transition to AD dementia. We also found decreases in the overlap of gray matter and amyloid connections mainly in medial temporal regions in CN Aβ+, which became more widespread in later disease stages in MCI Aβ + and AD Aβ + patients. Finally, our results pointed to a decrease of the multiplex brain hubs in MCI Aβ + and AD Aβ + patients, which were due to an imbalance in the number of connections in the two layers: specifically the gray matter layer showed lower connectivity in temporal regions and the amyloid layer showed higher connectivity in the anterior cingulate and cuneus in the Aβ + groups compared to CN Aβ−. Although these results may appear complex given that multiplex analyses have only been applied in a few studies in AD (Guillon et al. 2017; Yu et al. 2017; Guillon et al. 2019; Cai et al. 2020), they can be summarized into the following points: the overlapping connectivity strength and degree overlap detect changes across all stages of AD, whereas the other measures are sensitive to changes occurring in different disease stages such as the multiplex communities in MCI Aβ+ and the multiplex participation coefficient in MCI Aβ + and AD Aβ+. Our findings indicate that the interaction of amyloid deposition and gray matter atrophy within a multiplex approach across different disease stages shows information not provided by traditional graph theory approaches based on networks derived from single imaging modalities. Although we provided an interpretation to these different findings above, more studies are needed to understand better their biological meaning, which will become clearer when applied to larger and longitudinal samples in combination with other imaging modalities.

## Supplementary Material

Supplementary_Material_bhab429

## Notes

Data used in preparation of this article were obtained from the Alzheimer’s Disease Neuroimaging Initiative (ADNI) database (adni.loni.usc.edu). As such, the investigators within the ADNI contributed to the design and implementation of ADNI and/or provided data but did not participate in analysis or writing of this report. A complete listing of ADNI investigators can be found at: http://adni.loni.usc.edu/wp-content/uploads/how_to_apply/ADNI_Acknowledgement_List.pdf.
