# Supplementary material for: Multiplex connectome changes across the alzheimer’s disease spectrum using gray matter and amyloid data
Source: Cereb Cortex. 2022 Jan 20;32(16):3501–15. doi: 10.1093/cercor/bhab429 (PMC9376877; doi:10.1093/cercor/bhab429)
Supplement: Supplementary_Material_bhab429 [file supplementary_material_bhab429.zip › Supplementary_Material_bhab429.docx]

**Supplementary Material**

**“*Multiplex connectome changes across the Alzheimer’s disease spectrum using gray matter and amyloid data*” by Canal-Garcia et al.**

**Supplementary Table 1. Small-worldness network values obtained from the amyloid and gray matter layers in each group**

|  | **Amyloid layer** | | | | **Gray matter layer** | | | |
| --- | --- | --- | --- | --- | --- | --- | --- | --- |
| **Density** | **CN Aβ-** | **CN Aβ+** | **MCI Aβ+** | **AD Aβ+** | **CN Aβ-** | **CN Aβ+** | **MCI Aβ+** | **AD Aβ+** |
| **2%** | 15.59 | 61.62 | 23.90 | 24.38 | 5.53 | 29.13 | 31.95 | 18.21 |
| **3%** | 14.29 | 11.21 | 15.57 | 10.29 | 4.71 | 11.08 | 11.78 | 11.08 |
| **4%** | 6.58 | 6.96 | 6.99 | 6.25 | 4.30 | 6.11 | 4.89 | 5.28 |
| **5%** | 4.45 | 4.07 | 4.43 | 3.84 | 3.92 | 3.79 | 3.24 | 4.07 |
| **6%** | 3.06 | 3.68 | 3.76 | 3.14 | 3.23 | 3.09 | 2.30 | 3.36 |
| **7%** | 2.78 | 3.93 | 3.20 | 2.82 | 2.66 | 3.12 | 1.89 | 2.91 |
| **8%** | 3.10 | 2.81 | 2.63 | 2.75 | 2.45 | 2.60 | 2.07 | 2.76 |
| **9%** | 2.32 | 2.27 | 2.40 | 2.74 | 2.06 | 2.46 | 1.74 | 2.39 |
| **10%** | 2.17 | 2.06 | 2.58 | 2.73 | 1.93 | 2.39 | 1.68 | 2.07 |
| **11%** | 2.17 | 1.96 | 2.45 | 2.54 | 1.78 | 2.16 | 1.58 | 1.89 |
| **12%** | 1.94 | 1.97 | 2.46 | 2.56 | 2.07 | 2.09 | 1.65 | 1.78 |
| **13%** | 1.66 | 1.88 | 2.37 | 2.50 | 1.84 | 2.11 | 1.61 | 1.69 |
| **14%** | 1.45 | 1.91 | 2.22 | 2.42 | 1.58 | 1.96 | 1.46 | 1.39 |
| **15%** | 1.56 | 1.87 | 2.03 | 2.36 | 1.57 | 1.87 | 1.47 | 1.43 |
| **16%** | 1.47 | 1.76 | 1.83 | 1.77 | 1.59 | 1.68 | 1.56 | 1.39 |
| **17%** | 1.49 | 1.83 | 1.76 | 1.71 | 1.51 | 1.67 | 1.52 | 1.30 |
| **18%** | 1.44 | 1.74 | 1.68 | 1.74 | 1.43 | 1.42 | 1.45 | 1.23 |
| **19%** | 1.34 | 1.66 | 1.70 | 1.70 | 1.38 | 1.36 | 1.41 | 1.20 |
| **20%** | 1.32 | 1.63 | 1.65 | 1.63 | 1.34 | 1.33 | 1.40 | 1.13 |
| **21%** | 1.41 | 1.60 | 1.60 | 1.50 | 1.20 | 1.37 | 1.35 | 1.13 |
| **22%** | 1.36 | 1.69 | 1.57 | 1.47 | 1.19 | 1.27 | 1.29 | 1.12 |
| **23%** | 1.34 | 1.75 | 1.59 | 1.43 | 1.14 | 1.30 | 1.22 | 1.12 |
| **24%** | 1.32 | 1.74 | 1.55 | 1.41 | 1.18 | 1.33 | 1.19 | 1.11 |
| **25%** | 1.29 | 1.62 | 1.56 | 1.31 | 1.16 | 1.29 | 1.17 | 1.08 |
| **26%** | 1.29 | 1.62 | 1.52 | 1.30 | 1.15 | 1.31 | 1.15 | 1.07 |
| **27%** | 1.30 | 1.60 | 1.50 | 1.27 | 1.18 | 1.33 | 1.14 | 1.08 |
| **28%** | 1.33 | 1.58 | 1.50 | 1.27 | 1.16 | 1.33 | 1.12 | 1.11 |
| **29%** | 1.40 | 1.555 | 1.46 | 1.19 | 1.16 | 1.18 | 1.13 | 1.14 |
| **30%** | 1.29 | 1.07 | 1.35 | 1.21 | 1.17 | 1.14 | 1.14 | 1.11 |

All groups have a small-world index > 1 in both layers from density 2% to density 30%, indicating their amyloid and gray matter network have small-world properties.

**Supplementary Figure 1. Regional gray matter and amyloid values in each group**

**
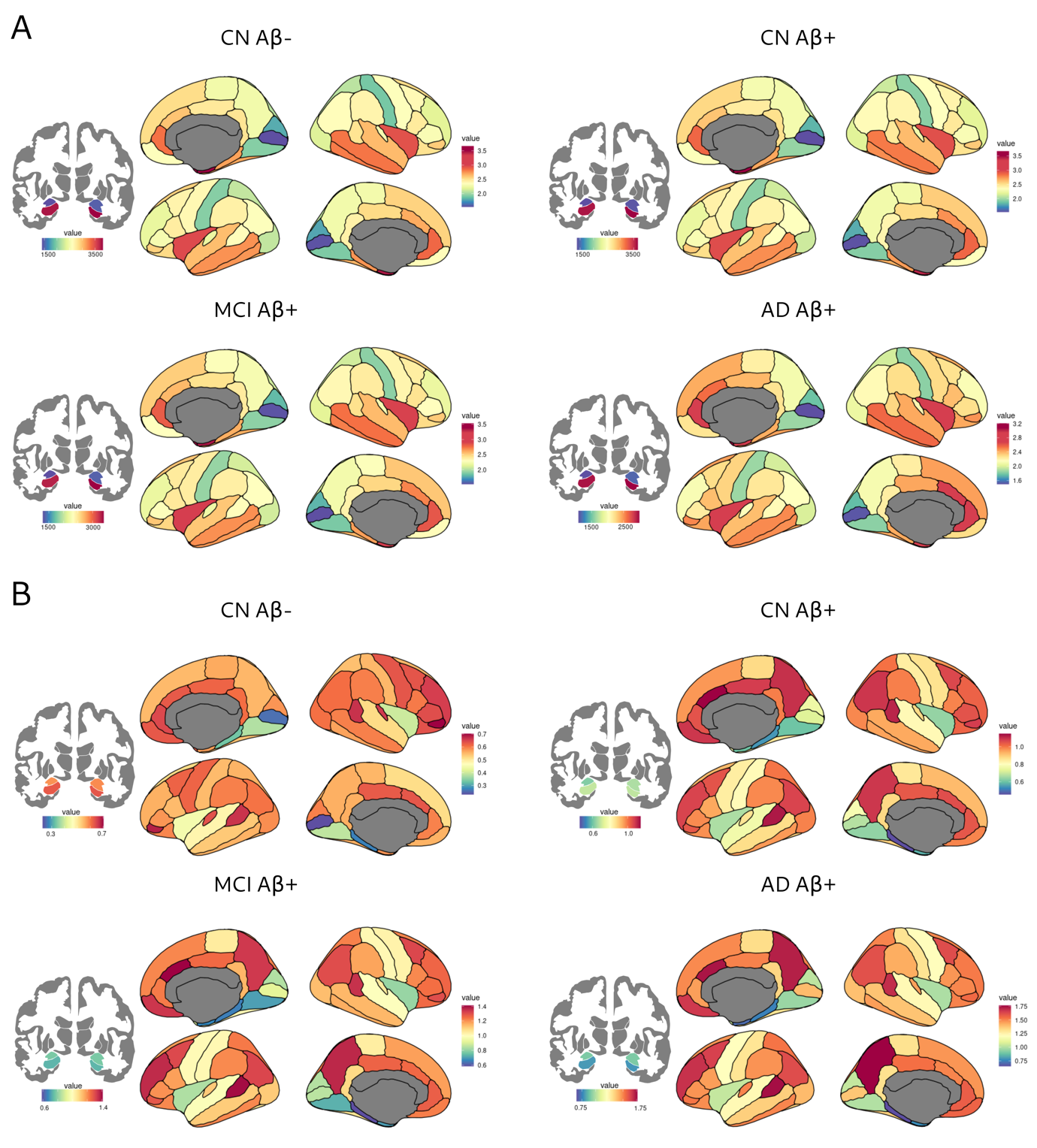
**

Mean cortical thickness values in the 68 cortical regions and mean volumes in the 4 subcortical regions (A), mean ^18^F-florbetapir PET values (B) in the 72 cortical and subcortical regions in cognitively normal (CN) subjects without amyloid-β pathology (Aβ-) in addition to CN, patients with mild cognitive impairment (MCI) and patients with Alzheimer’s disease (AD) dementia with amyloid-β pathology (Aβ+). Regions colored in warmer colors represent higher regional gray matter values or amyloid values, whereas regions colored in colder colors represent lower regional gray matter/amyloid values.

**Supplementary Figure 2. Normalized Variation of Information matrices for each group with all combinations of gamma and omega for the detection of multiplex modules**

**
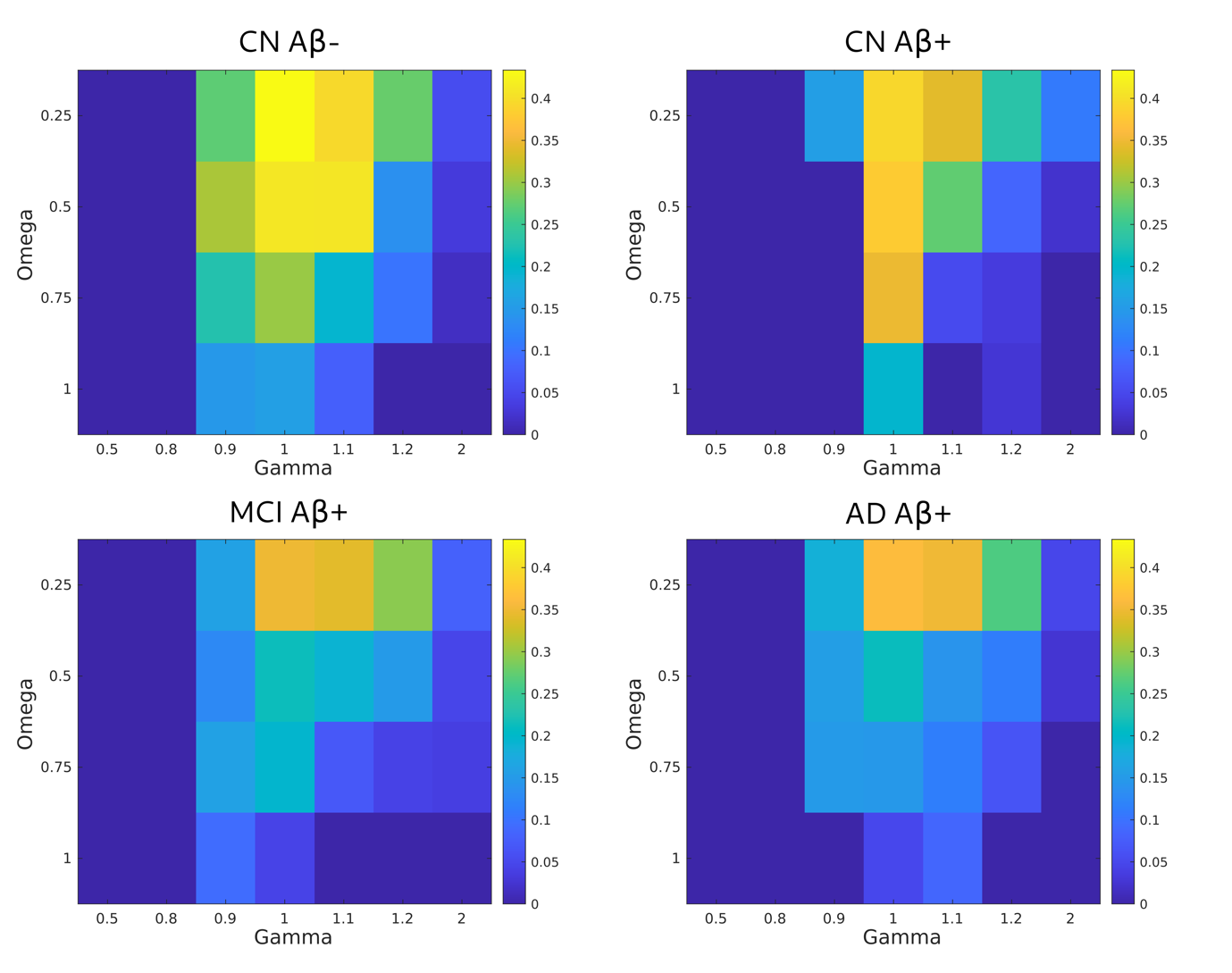
**

Variation of Information (VI) matrices calculated for each combination of gamma and omega in cognitively normal (CN) subjects without amyloid-β pathology (Aβ-) in addition to CN, patients with mild cognitive impairment (MCI) and patients with Alzheimer’s disease (AD) dementia with amyloid-β pathology (Aβ+). Each entry of the normalized VI group matrix corresponds to the distance between the community assignments from each layer at a certain combination of parameters.

**Supplementary Figure 3. Weighted matrices of each group**

**
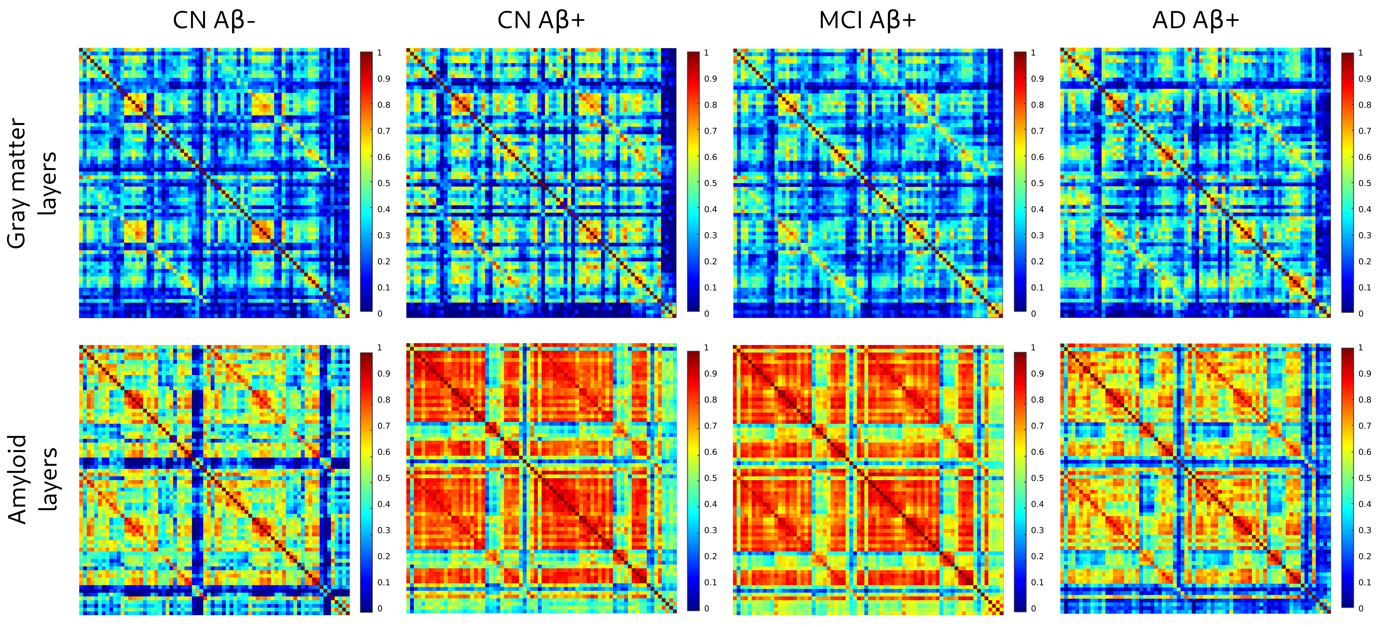
**

Group weighted correlation matrices for each layer in cognitively normal (CN) subjects without amyloid-β pathology (Aβ-) in addition to CN, patients with mild cognitive impairment (MCI) and patients with Alzheimer’s disease (AD) dementia with amyloid-β pathology (Aβ+). In the gray matter layer, the nodes were defined using the mean cortical thickness or subcortical volumes of 72 brain regions, whereas in the amyloid layer the nodes were defined using the mean SUVR values of the same brain regions. We can observe that the amyloid matrices have stronger connections than the gray matter matrices.

**Supplementary Figure 4. Regional degree overlap in each group**

**
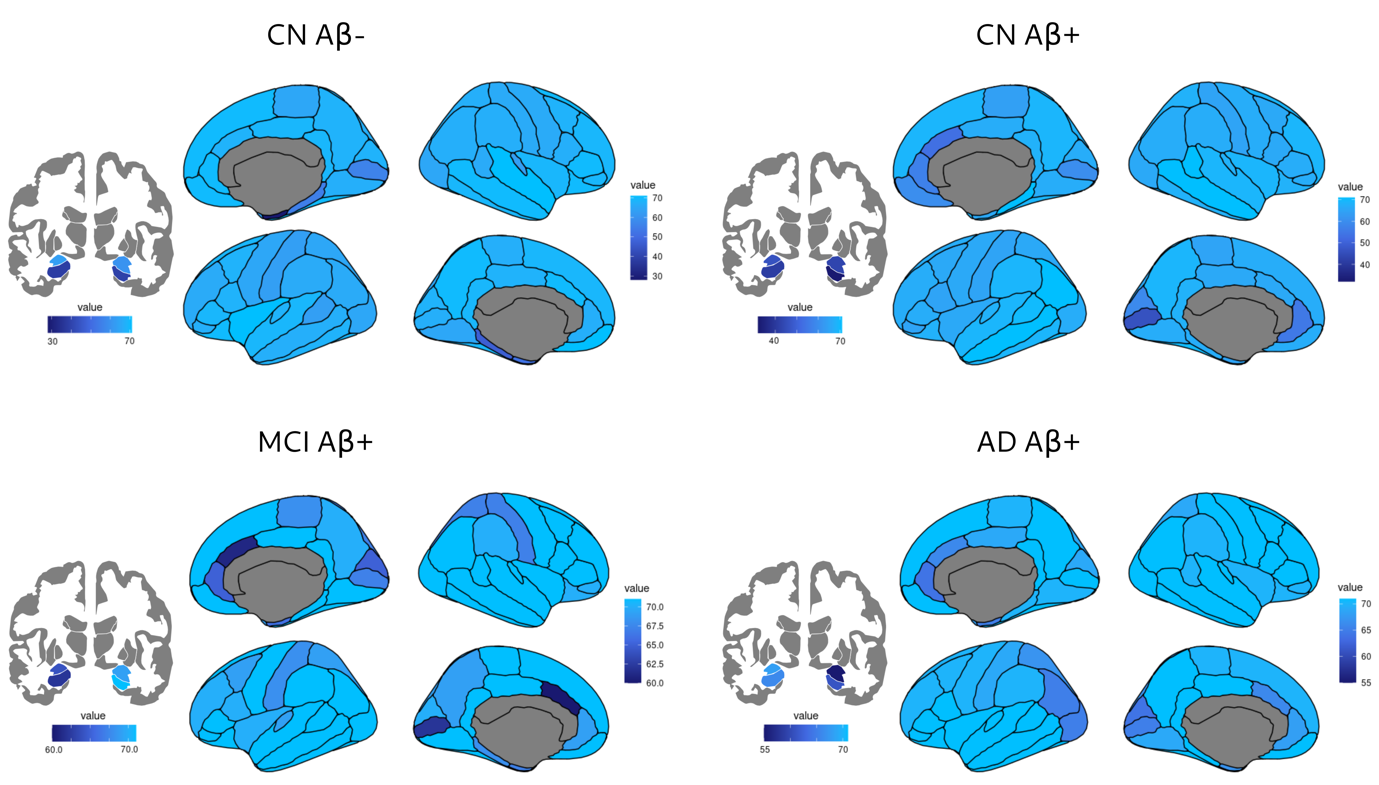
**

Values of the degree overlap in cognitively normal (CN) subjects without amyloid-β pathology (Aβ-) in addition to CN, patients with mild cognitive impairment (MCI) and patients with Alzheimer’s disease (AD) dementia with amyloid-β pathology (Aβ+).

**Supplementary Figure 5: Secondary multiplex analysis with cortical volumes instead of cortical thickness**

**
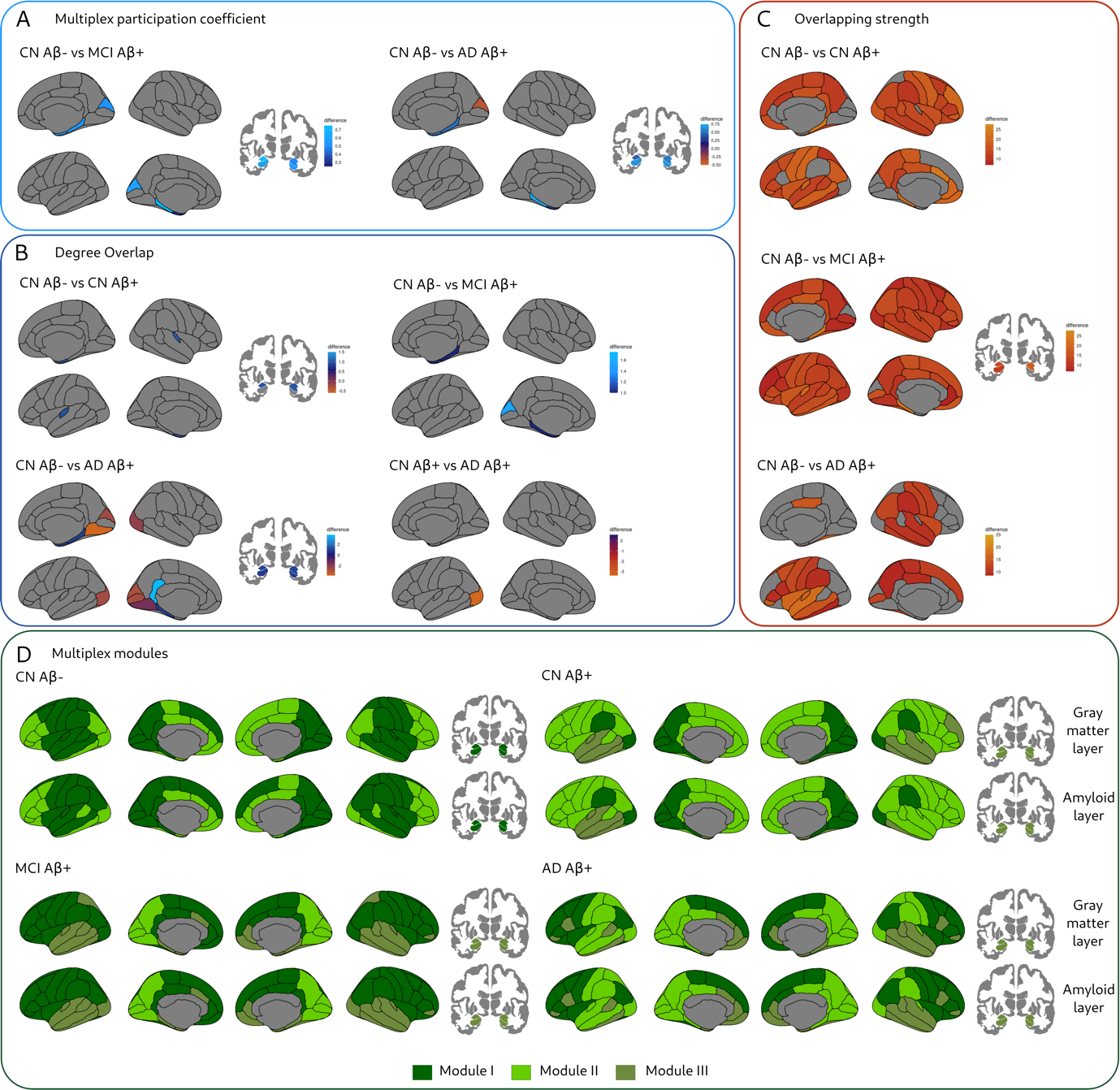
**

Results of the multiplex participation coefficient (A), degree overlap (B), overlapping strength (C), and multiplex communities (D) using cortical volumes instead of cortical thickness to build the first layer in cognitively normal (CN) subjects without amyloid-β pathology (Aβ-) in addition to CN, patients with mild cognitive impairment (MCI) and patients with Alzheimer’s disease (AD) dementia with amyloid-β pathology (Aβ+).
